# A Therapeutic Proposal for Mini-Puberty in Male Infants with Hypogonadotropic Hypogonadism: A Retrospective Case Series

**DOI:** 10.3390/jcm13226983

**Published:** 2024-11-20

**Authors:** María Aurora Mesas-Aróstegui, Fidel Hita-Contreras, Juan Pedro López-Siguero

**Affiliations:** 1 Pediatric Endocrinology Department, Instituto Hispalense de Pediatría, Hospital Quirón Marbella, 29603 Málaga, Spain; 2 Pediatrics Department, Hospital of Guadix, 18500 Granada, Spain; 3 Department of Health Sciences, Faculty of Health Sciences, University of Jaén, 23071 Jaén, Spain; 4 Pediatric Endocrinology Department, Hospital Regional Universitario de Málaga, 29010 Málaga, Spain

**Keywords:** congenital hypogonadotropic hypogonadism, micropenis, cryptorchidism, mini-puberty, gonadotropins, testosterone

## Abstract

**Background:** Male patients with congenital hypogonadotropic hypogonadism (CHH) have impaired postnatal activation of the hypothalamic–pituitary–gonadal axis that occurs during mini-puberty. The aim of this study was to report our experience using gonadotropin replacement therapy for mini-puberty in male infants with CHH and to establish treatment recommendations. **Methods:** The patients included in this retrospective case series (*n* = 9) were diagnosed in the postnatal period due to micropenis, with two being accompanied by cryptorchidism and four with other associated hormonal deficits. All patients started treatment with gonadotropins early after diagnosis, between 2 weeks and 5 months of age, with a schedule of discontinuous injections with subcutaneous human chorionic gonadotropin (62.5–500 IU) two times per week and recombinant follicle-stimulating hormone-alpha (37.5–75 IU) three times per week. **Results**: The data from our study show an early response, ranging from almost undetectable levels of testosterone at diagnosis to elevated levels after starting treatment, as well as a positive clinical response with increases in testicular volume and penis size in all cases without requiring complementary treatment with testosterone esters and without adverse effects. **Conclusions:** Our results show that gonadotropin replacement therapy is a well-tolerated and effective treatment for testicular and penile problems in male patients with CHH.

## 1. Introduction

The hypothalamic–pituitary–gonadal (HPG) axis is activated three times during life: in the second trimester of pregnancy, during mini-puberty (a period that starts a few days after birth and ends at about 6 months of life in males), and during puberty. Symptoms, biochemical findings, and the influence of different related genes vary depending on the stage. After birth, the HPG axis is transiently activated [1], producing an increase in gonadotropin levels under normal conditions, which remain elevated until about 6 months of age in males, leading to an increase in testosterone, insulin-like 3 peptide (INSL-3), inhibin B, and anti-Müllerian hormone (AMH) [2,3] while stimulating genital virilization and future fertility. Gonadotropin and testosterone reach maximum levels between the first and third months of life, achieving similar levels to those in the postpubertal period and stimulating penile growth that can continue until 3 years of age [4]. In addition, it has been shown that the presence of elevated testosterone levels at this time is related to male neurobehavioral development [5,6,7]. On the other hand, elevated follicle-stimulating hormone (FSH) levels are positively correlated with testicular growth due to increases in the total numbers of germ cells and Sertoli cells [3]. Thereafter, testosterone levels will gradually decrease until 6 months of age, remaining at almost undetectable levels until puberty [1,2]. According to previously published data, this activation follows dynamic behavior based on a two-phase activation process. The initial stage starts with increased Leydig cell activity, takes place in the first and third months, and continues to the second stage with the subsequent activation of Sertoli cells around the fourth month of life [8].

Furthermore, 5 alpha-dihydro-testosterone (DHT) has a very important role in the masculinization of the external genitals in males through both the classical androgenic pathway and an alternative (backdoor) pathway. The integrity of this backdoor pathway is necessary for the normal development of a male fetus. Androsterone seems to be the predominant androgen of this backdoor pathway, with placental progesterone being its most likely substrate [9].

Hypogonadotropic hypogonadism (HH) occurs due to hypothalamic–pituitary insufficiency, resulting in the poor secretion of gonadotropins, luteinizing hormone (LH), and/or FSH. Its etiology is diverse and can be of hypothalamic or pituitary origin, and it is congenital or acquired, permanent or transient, and isolated or associated with other hormonal defects; additionally, it can present with different severities. It is traditionally considered a predominantly male condition with a male/female gender ratio of 3.6:1, a situation that remains unexplained [10]. Familial forms are in the minority, and different patterns of inheritance have been observed, including autosomal dominant, autosomal recessive, and X-linked recessive, while there is evidence of oligogenic inheritance in up to 20% of cases [11]. Although it is generally considered a rare disorder, some retrospective studies conducted in Nordic countries estimated that the prevalence of male congenital hypogonadotropic hypogonadism (CHH) is 1 in 4415–15,000 [12].

In recent years, several international publications have explored mini-puberty replacement therapy in patients with gonadotropins in different modalities [13,14] to allow for more adequate gonadal development and secretion of sex steroids, which could theoretically restore reproductive capacity in these patients. In addition, early treatment in mini-puberty seems to prepare the testicles for later induction at puberty [14,15].

The aim of combined therapy with gonadotropins in the absence of mini-puberty is to achieve normal levels of testosterone, inhibin B, and AMH to correct the micropenis and/or cryptorchidism or to prepare the testis, creating a more favorable environment (i.e., the secretion of inhibin B, proliferation of germ cells, or testicular growth) to stimulate a better response after combined therapy with gonadotropins at puberty [14].

Using recombinant FSHs during mini-puberty would induce an increase in testicular volume through the proliferation of Sertoli cells, producing inhibin B and AMH, while using human chorionic gonadotropin (hCG) would stimulate the Leydig cells, increasing the secretion of testosterone and INSL3 [15,16]. Treatment with gonadotropins is safe at this age because, although it may increase intratesticular gonadotropin concentrations, it would not stimulate spermatogenesis or adversely affect the number of Sertoli cells, as they do not express the androgen receptor until 5 years of age [17].

This case series was conducted to assess the results of gonadotropin replacement therapy in a group of infants with congenital hypogonadotropic hypogonadism during mini-puberty, evaluating clinical and biochemical responses with the aims of mimicking physiological male mini-puberty, resolving cryptorchidism and micropenis, and restoring the function of Leydig and Sertoli cells. It determines the most appropriate and safest dose of gonadotropins and the most appropriate duration of treatment in this group of patients.

## 2. Materials and Methods

### 2.1. Study Design and Participants

This was a retrospective observational case series conducted on a cohort of nine patients with CHH diagnosed in the newborn or infant period. This study was carried out in the Pediatric Endocrinology Department of a tertiary hospital in Spain (Hospital Regional Universitario de Málaga), and patients were included between January 2017 and December 2023. This research was conducted in accordance with the Declaration of Helsinki, good clinical practices, and all applicable laws and regulations.

The inclusion criteria were as follows: a CHH diagnosis based on clinical and laboratory findings with the presence of micropenis (penis size < −2.5 SD) and/or cryptorchidism in the absence of normal mini-puberty, along with decreased levels of LH (<0.3 UI/mL) and testosterone (<0.24 ng/mL).

### 2.2. Outcomes

Brain MRI (magnetic resonance imaging), karyotype, and genetic panel were performed on all patients except one (for whom they have still not been performed).

Penile length was assessed with a rigid ruler, and testicular size was assessed with the Prader orchidometer. LH serum concentrations and testosterone levels were measured using radioimmunoassay. All measurements were performed by specialists from the Hospital Regional Universitario de Málaga (Spain).

The follow-up sessions were conducted with clinical and analytical evaluations one month after starting treatment and subsequently every two or three months until the treatment was completed. Testosterone values were monitored at the first-, third-, and sixth-month mark after beginning treatment and again at the end of treatment (between 8 and 12 months of age).

### 2.3. Intervention

All patients started the treatment shortly after diagnosis. It consisted of hCG administration twice per week and recombinant FSH-alpha (rFSH-alpha) administration three times per week, together in the same syringe, in concentrations of 62.5 to 500 IU/dose for hCG and 37.5 to 75 IU/dose for rFSH-alpha. The treatments were maintained until around 6 months of age.

### 2.4. Statistical Analysis

A statistical analysis was performed using SPSS software (version 20.0, SPSS Inc., Chicago, IL, USA). The level of statistical significance was set at a *p*-value < 0.05. The normality of the data distribution was evaluated using the Shapiro–Wilk test. The variables are presented as the median and interquartile range (percentile 25–percentile 75). The nonparametric Wilcoxon signed-rank test was employed to evaluate the changes in penis size and testicular volume before and after the treatment and due to the non-normality of the data. The effect size (r) was calculated by dividing the z value by the square root of the sample size.

## 3. Results

Table 1 shows the baseline data in order of the date of diagnosis. The patients treated in our study had different etiological origins. Four patients had panhypopituitarism, presenting with other associated hormonal deficits. Another four patients presented isolated CHH, with one patient presenting syndromic features and another one presenting Prader–Willi syndrome. All patients had adequate weights and lengths for their gestational age except for the last patient (small for their gestational age). Two patients (with Prader–Willi syndrome and who were small for their gestational ages) presented with cryptorchidism. In both cases, the testes descended to the scrotum at the end of the treatment. All patients with panhypopituitarism presented ectopic neurohypophysis in brain MRI, two of them presented with pituitary hypoplasia, and a patient considered small for their gestational age presented with a flattened Turkish saddle with an altered neurohypophysis and lower brightness at T1-weighted MRI.

The karyotype was 46 XY in all cases. The next-generation sequencing CHH gene panel was negative in four patients. Patients with syndromic features presented with variant of uncertain significance in heterozygosity in CLCN7, OPTA2, OPTA4, SOX9, and ROR2. The results for the last patient included in this review are pending. The patient with Prader–Willi syndrome presented with maternal uniparental disomy on chromosome 5 and a mutation of uncertain significance in heterozygosity in KBTBD13 (c.1153C›T).

The median age at the beginning of treatment was 1.50 months (0.85–2.25). The median LH levels at diagnosis were 0.17 (0.07–0.85) mIU/mL, and the testosterone levels went from an almost undetectable level with a median of 0.07 (0.04–0.16) ng/mL to supraphysiological levels after the first laboratory control after treatment initiation with a median of 9.85 (6.69–12.67) ng/mL. The values ranged from 5.41 ng/mL in the patient classified as small for their gestational age to a maximum value of 15 ng/mL. These values are shown in Table 2. The last patient recently completed treatment, so their post-treatment data could not be provided.

As for the hCG, the initial treatment doses were administered according to previously published studies, starting with 250 IU of hCG twice per week, except one patient who started treatment earlier (2 weeks of age). This patient started with 500 IU, but after the first analytical control, the dose was reduced to 250 IU due to a testosterone level of 14.56 ng/mL. A lower dose, namely 62.5 IU, was used in the last three patients due to difficulties in accessing previously used dosages.

The initial treatment dose of rFSH-alpha was 37.5 IU three times per week in all patients except the younger patient (the first in this series), who started treatment earlier, and received an initial dose of 75 IU (after the first laboratory control, this was reduced to 37.5 IU).

After the first clinical control, all patients presented an increase in testicular size (*p*-value = 0.005), which was initially less than the range of 1 cc to 2 cc (Table 3), except for the patient with Prader–Willi syndrome who was small for their gestational age, who presented a test size near 1.5 cc. Cryptorchidism was resolved in both patients, although, in the patient with Prader–Willi syndrome, one of their testicles experienced a partial descent, staying at the end of the canalis inguinalis. The effect size can be considered as high (r = 0.937).

The median penis size was 20 mm (18.00–26.50) in length × 6 mm (6–8) in width, and the smallest penis size was observed in the patient who was small for their gestational age, with a penile size of 12 mm in length × 6 mm in width. Finally, after the treatment, the penile size increased to 44 mm (40–46) in length (*p*-value = 0.008) × 13 mm (12.50–14.00) in width (*p*-value = 0.011). The effect size was high for both penis length (r = 0.889) and width (r = 0.853). The data are shown in Table 3.

The median treatment time was 3.5 months. None of the patients had local or side effects, and the treatment was well accepted and supported by their families.

## 4. Discussion

The objective of this study was to evaluate the beneficial effects of treatment with gonadotropins during mini-puberty in patients with congenital hypogonadotropic hypogonadism. The results of this case study show that gonadotropin replacement therapy improves endocrine testicular function, penis size, and testicular volume and offers the possibility of solving cryptorchidia in these patients.

Undervirilization signs and micropenis usually result from hormone-related disturbances in the development of the male reproductive and genitourinary systems. The main causes are hypogonadotropic hypogonadism due to pituitary or hypothalamic insufficiency, hypergonadotropic hypogonadism due to primary testicular insufficiency, and idiopathic hypogonadotropic hypogonadism [18].

Congenital hypogonadotropic hypogonadism refers to an infrequent situation that occurs in different etiological processes, resulting in a heterogeneous group in which it is difficult to compare cases. Mini-puberty replacement therapy tries to mimic physiological mini-puberty in these cases [19].

According to the results published by Busch et al. [20] in 2022 in the COPENHAGEN Mini-Puberty Study performed with a cohort of 128 healthy neonates, gonadotropins rise to a maximum peak a few weeks after birth and target cells have a sequential activation. Maximum testosterone and INSL3 increases occur in the first month of life, while AMH and inhibin B increases take place during the first 4–5 months of age, showing the importance of starting treatment as soon as possible. In addition, it is well documented that one of the best predictors for future spermatogenesis is testicular size [21], so a better physiological approach is using gonadotropin replacement therapy to treat patients with HCC, inducing mini-puberty at the right time, which could lead to a real improvement in reproductive capacity in adulthood [21,22].

The first study on this topic was published by Main et al. in 2002 [23], which was based on a 7.9-month-old infant diagnosed with CHH and treated for about 6 months with two weekly injections of recombinant LH (rLH) and rFSH. Since then, several publications showing their own experiences in treating infants with CHH have appeared [24,25,26,27,28,29,30,31] that demonstrate that replacement therapy has a positive effect on penile enlargement, testicular volume increase, and the restoration of hormone levels in this period in all cases, although there are still no published long-term results. Table 4 summarizes the most important results of these studies.

In 2019, Papadimitriou et al. presented the first results of the REMAP (replacement of male mini-puberty) study [28], the first longitudinal study with up to ten years of follow-up data for some of the patients. In this study, ten patients with CHH received a daily subcutaneous injection of fixed doses of rLH/rFSH 75/150IU (Pergoveris^®^) for 3 months. They described improvements in testosterone, inhibin B, and AMH levels during the intervention and an increase in the mean penile mean length from 2 to 3.8 cm. During therapy, testicular descent to the scrotal position was seen in all cases, although two of them required subsequent orchidopexy due to testicular regression at the inguinal level and three required additional treatment with testosterone enanthate. There are limited studies from Spain, although one publication involves two case series reporting treatment experiences with the first two patients who were part of this study [30].

Most previously published studies used rLH and rFSH as replacement therapy, administered either using a continuous infusion pump or through multiple weekly injections, but a few used hCG to induce mini-puberty. One of these studies is that by Avril et al. [31] where they compared a group treated with injections of hCG + FSH (260 IU two times per week and 25 IU three times per week) for 3 months versus a group treated with a continuous pump (FSH 75 + LH 75/day) for 6 months. According to their results, slightly higher testosterone values were observed with the injection group compared to the pump group, and this could be due to the longer average life of hCG with respect to rLH.

In the present study, nine patients were diagnosed in the newborn/infant period, starting early with gonadotropin treatment based on weekly injections. The treatment doses were initially established by previously published studies and were empirically adjusted according to our own observations. The chosen intervention was rFSH-alpha (Gonal F^®^) three times per week (Monday, Wednesday, and Friday) and hCG (Gonasi^®^) twice per week (Monday and Friday) with the aim of achieving increases in the Sertoli cell mass, testicular volume, and testosterone and INSL3 secretion by stimulating the Leydig cells [21,22]. We used hCG in our population because it is easier to access, and we used this formulation in our service. This is supported by knowledge of the steroidogenic role of Leydig cells, which express luteinizing hormone/choriogonadotropin receptor (LHCGR) but not the FSH receptor (FSHR); additionally, given that hCG and LH use the same receptor, although evidence is still limited, some studies point to hCG having a higher binding affinity than LH due to its greater persistence at the receptor level and its longer average hormone binding time [32]. Therefore, with two weekly administrations of hCG, we achieved adequate testosterone levels and resolved cryptorchidism with a positive and early response in all cases without the need to complete subsequent treatments with testosterone esters.

Gonadotropin replacement therapy is generally well tolerated, and some of the observed adverse effects, such as acne or gynecomastia, are infrequent and mostly reported in male adolescents and adults [33]. Although Main et al. (2002) reported recurrent otitis media, sleep disturbances, intermittent nausea, and a local rash once at the injection site (spontaneously resolved) in treatment during mini-puberty [23], the literature demonstrates that negative effects were rarely found in infants; however, these results should be taken with caution due to the small number of studies involving infants treated during mini-puberty [34]. No adverse effects or local rash at the injection site were observed in the present study. In addition, the use of this intervention with multiple injections has been well accepted by the patients’ families because specific training is not required (as with the use of a continuous infusion pump). Therefore, in our experience, replacement therapy with rFSH and hCG is effective and efficient.

As we previously mentioned, one of the targets of mini-puberty replacement therapy is to create a more favorable environment to promote a better response after combined therapy with gonadotropins in the puberty period [14,15]. Stuckey et al. [35] recently published a very interesting article describing their experience with a patient with Kallman syndrome. A CHH diagnosis was made due to the presence of micropenis and bilateral cryptorchidism, and the patient received treatment for 2 months within the first year of life with a pulsatile pump administration of GnRH. A positive hormonal and clinical response and bilateral testicular descent were initially observed, although they required right orchidopexy at 21 months. Puberty was induced with the classic intervention with testosterone esters, and, subsequently, the patient continued receiving quarterly injections of testosterone undecanoate. At the age of 33 years, treatment with 1500 IU of hCG and 150 IU of rFSH three times per week was proposed, presenting a considerable increase in testicular volume and a total sperm count of 25.5 million after 12 months of treatment, allowing the patient to achieve natural conception with their partner in life.

Based on our experience, our suggested approach is to initiate the treatment with a dose of rFSH-alpha 37.5 IU three times per week, which can be increased to 75 IU, and a hCG dose of 62.5 IU twice per week, which can be increased to up to 250 IU/dose if necessary, depending on the serum testosterone concentrations one month after the beginning of treatment. Then, it can be administered every 2 months until the end of the treatment. Based on our observations, the treatment should be established as soon as possible, and a duration between 2 and 3 months may be sufficient to achieve treatment goals.

The possibility of reaching supraphysiological testosterone levels during treatment should not be a reason to avoid treatment, as high concentrations of intratesticular gonadotropins would not stimulate spermatogenesis or negatively affect the Sertoli cell count since the androgen receptor is not expressed in these cells until the fifth year of life [17].

The limitations of this study include the small number and heterogeneity of the patients. We need to continue investigating the possible long-term positive effects of gonadotropin replacement therapy and specific nonreproductive consequences at the metabolic, cardiovascular, neurological, and behavioral levels. Therefore, it is important to promote the creation of an international and prospective registry that allows for a homogeneous treatment to be offered to these patients. In this context, a very interesting article recently published by Rhys-Evans et al. [34] presents a specific electronic record of patients with HCC, and it seems to be a good basis to continue advancing the knowledge that allows us to offer better options to our patients and their families.

## 5. Conclusions

Treatment with gonadotropins to replace male mini-puberty is stimulating and could cause a real change in the lives of these patients. Although more studies are needed to evaluate the possible long-term effects, in our own experience, carrying out gonadotropin replacement therapy during mini-puberty is safe, well tolerated by patients, and allows for testicular endocrine function to be restored (by improving penis and testicular size) through the proper secretion of testosterone. It also has the potential to resolve cryptorchidism, allowing for surgical correction to be avoided in most cases. This treatment opens the door to possible fertility function for patients with CHH, an inaccessible option until now. The possible long-term positive effects of gonadotropin replacement treatment on adequate physical development, psychological well-being, and cardiovascular and metabolic health still need to be studied.

## Figures and Tables

**Table 1:** Clinical, MRI, and genetic findings.

Patient Number	Clinical Features	Diagnosis	Karyotype/Molecular Genetic Study	Brain MRI Findings
1	Micropenis	Panhypopituitarism	46XY/negative	Pituitary hypoplasia, ectopic neurohypophysis
2	Micropenis	Panhypopituitarism	46XY/negative	Pituitary hypoplasia, ectopic neurohypophysis
3	Micropenis	CHH	46XY/negative	No findings
4	Micropenis	CHH Syndrome	46XY/VUS in CLCN7, OPTA2 and OPTA4, SOX9 and ROR2 heterozygosity	No findings
5	Micropenis	CHH	46XY/negative	No findings
6	Micropenis	Panhypopituitarism	46XY/negative	Ectopic neurohypophysis with (probable) infundibular agenesis
7	Micropenis	Panhypopituitarism	46XY/negative	Ectopic neuropituitary gland
8	Cryptorchidism	Prader–Willi Syndrome	A 46XY/maternal uniparental dysomy chromosome 5/mutation in heterozygosity in KBTBD13 c.1153C›T	Still not performed
9	Micropenis and Cryptorchidism	CHH	46XY/extracted	Flattened Turkish saddle, normal adenohypophysis, neuropituitary gland with decreased brightness at T1

CHH: congenital hypogonadism hypogonadotropic; MRI: magnetic resonance imaging. VUS: variant of uncertain significance.

**Table 2:** LH and testosterone serum levels at diagnosis, during treatment, and after treatment.

Patient		Diagnosis		During Treatment	After Treatment
	Age at the Beginning of Treatment (Months)	LH (UI/L)	Testosterone (ng/mL)	Testosterone (ng/mL)	Testosterone (ng/mL)
1	1.5	0.1	0.03	14.56	<0.07
2	0.5	0.54	0.04	6.62	0.03
3	2.5	1.15	0.038	9.61	<0.07
4	1.5	1.83	0.23	9.85	<0.07
5	1	0.07	0.07	6.75	<0.07
6	0.7	0.07	0.08	15	<0.07
7	2	0.07	0.07	9.9	<0.07
8	5	0.17	0.07	10.77	<0.07
9	2	0.34	0.26	5.41	

LH: luteinizing hormone.

**Table 3:** Penis and testicle sizes at diagnosis and after treatment.

Patients.	Penis Size (Length × Circumference, mm)		Testicular Size (cc)	
	Diagnosis	After Treatment	Diagnosis	After Treatment
1	18 × 5	45 × 12	1	2
2	20 × 6	42 × 14	<1	2
3	19 × 8	38 × 13	<1	2
4	21 × 6	47 × 16	1	2
5	18 × 6	42 × 13	<1	2
6	25 × 6	44 × 13	<1	2
7	28 × 8	45 × 14	<1	2
8	33 × 8	49 × 13	<1	1.5
9	12 × 6	33 × 10	<1	1.5

**Table 4:** Summary of previous published experiences.

Authors/Year of Publication/ Type of Study	Patients	Age at Start of Treatment (Months)	Intervention	Duration of Treatment (Months)	Clinical Outcomes	Biochemical Outcomes
Main et al. [23] 2002 One case series	1	7.9	Two weekly injections with rLH (20–40 IU) and rFSH (2.5 IU/Kg)	5.8	Increase in penis length from 1.6 cm to 2.4 cm; increased TV requiring subsequent treatment with testosterone enanthate.	Increased LH, FSH, and inhibin B to normal limits; testosterone levels remained undetectable.
Bougnères et al. [24] 2008 Two case series	2	P1: 2 P2: 5	Continuous pump infusion P1: 56 IU rhLH and 67 IU rhFSH/day; P2: 50 IU rhLH and 125 IU rFSH/day	P1: 4.2 P2:7	TV increased from 0.45 to 0.57 mL at birth to 2.10 mL at 7 months; length of stretched penis increased from 8 to 30 mm (P1) and from 12 to 48 mm (P2).	LH and FSH at normal or supranormal levels; increased testosterone levels in normal range.
Sarfati et al. [25] 2015 One case series	1	1	Continuous pump infusion with 75 IU rLH and 75 IU rFSH/day	7	Increases in TV (from 0.33 mL to 2.3 mL) and penile length (15 to 38 mm).	Increased gonadotropins, inhibin B, and testosterone.
Lambert and Bougnères [26] 2016 Retrospective descriptive study	8	6.03 ± 3.75 (0.25 to 11)	Continuous pump infusion with 50 IU rhLH and 75 IU rhFSH	6 ± 0.58 months	Increased TV in all patients and increase in penile length from 2.02 cm to 3.74 cm.	Increased serum gonadotropins and testosterone to normal levels.
Stoupa et al. [27] 2017 Retrospective descriptive study in French tertiary education center	6	3-5.5	Continuous pump infusion with 150 IU/day rLH and 75 IU/day rFSH	4–5	Increase in penile length from 13 to 38 mm in all patients except one patient with partial androgen insensitivity syndrome.	Testosterone increased from undetectable levels to 3.5 ± 4.06 ng/mL.
Papadimitriou et al. [28] 2019 Longitudinal descriptive study with up to 10 years of follow-up	10	2.3–9.4	Daily injection with combined rLH/rFSH 75/150 IU	3	Increase in penile length from 2 to 3.8 cm; patients with cryptorchidism improved during therapy, but two cases required surgical correction and three patients required complete treatment with testosterone enanthate.	Increased LH, FSH, and testosterone to normal levels.
Kohva et al. [29] 2019 Retrospective review at three tertiary institutions in Finland between 2006 and 2016	5	0.7–4.2	rhFSH (3.4 IU/kg–7.5 IU/kg 2 or 3 times per week) + testosterone (25 mg im monthly)	3–4.5	Increase in penile length by 81 ± 50%; all patients with cryptorchidism required orchidopexy at 2.0 ± 0.7.	Increased inhibin B levels from 76 ± 18 ng/L to 176 ± 80 ng/L.
Álvarez Casaño and López Siguero. [30] 2019 Two case series	2	P1: 1.5 P2: 0.5	rhFSH (37.5–75 IU/dose) 3 times per week + hCG (500–250 IU/dose) 2 times per week	P1: 6 P2:7	Increases in penile length (40 × 11 mm and 42 × 14 mm) and testicular volume to 2 cc.	Testosterone increased from undetectable to supraphysiological levels.
Avril et al. [31] 2023 Multicenter retrospective study in two tertiary institutions in Paris comparing results according to pump administration vs. injection between 2004 and 2019	35	Mean of 5.1 months in pump-treated group and 13 months in the multiple injection group	Eighteen patients receiving continuous pump infusion with 150 IU/day rLH and 75 IU/day rFSH 17 patients receiving weekly injections with hCG and FSH	Six months for pump group; three months for multiple injection group	Significantly greater increase in penile length in injection group compared to pump group; improvement in testicular descent in both groups.	Significant increases in testosterone, AMH, and inhibin B levels were observed in both groups.

Abbreviations: FSH: follicle-stimulating hormone; LH: luteinizing hormone; hCG: human chorionic gonadotropin; im: intramuscular; P: patient; rLH: recombinant LH; rFSH: recombinant FSH; rhFSH: recombinant human FSH; TV: testicular volume.

## Data Availability

The original contributions presented in the study are included in the article, further inquiries can be directed to the corresponding authors.

## References

[1] Renault C.H., Aksglaede L., Wøjdemann D., Hansen A.B., Jensen R.B., Juul A. (2020). Minipuberty of human infancy—A window of opportunity to evaluate hypogonadism and differences of sex development?. Ann. Pediatr. Endocrinol. Metab..

[2] Becker M., Hesse V. (2020). Minipuberty: Why Does it Happen?. Horm. Res. Paediatr..

[3] Lucaccioni L., Trevisani V., Boncompagni A., Marrozzini L., Berardi A., Iughetti L. (2021). Minipuberty: Looking Back to Understand Moving Forward. Front. Pediatr..

[4] Bay K., Virtanen H.E., Hartung S., Ivell R., Main K.M., Skakkebaek N.E., Andersson A.M., Toppari J., Nordic Cryptorchidism Study Group (2007). Insulin-like factor 3 levels in cord blood and serum from children: Effects of age, postnatal hypothalamic-pituitary-gonadal axis activation, and cryptorchidism. J. Clin. Endocrinol. Metab..

[5] Boas M., Boisen K.A., Virtanen H.E., Kaleva M., Suomi A.M., Schmidt I.M., Damgaard I.N., Kai C.M., Chellakooty M., Skakkebaek N.E. (2006). Postnatal penile length and growth rate correlate to serum testosterone levels: A longitudinal study of 1962 normal boys. Eur. J. Endocrinol..

[6] Lamminmäki A., Hines M., Kuiri-Hänninen T., Kilpeläinen L., Dunkel L., Sankilampi U. (2012). Testosterone measured in infancy predicts subsequent sex-typed behavior in boys and in girls. Horm. Behav..

[7] Pasterski V., Acerini C.L., Dunger D.B., Ong K.K., Hughes I.A., Thankamony A., Hines M. (2015). Postnatal penile growth concurrent with mini-puberty predicts later sex-typed play behavior: Evidence for neurobehavioral effects of the postnatal androgen surge in typically developing boys. Horm. Behav..

[8] Busch A.S., Ljubicic M.L., Upners E.N., Fischer M.B., Raket L.L., Frederiksen H., Albrethsen J., Johannsen T.H., Hagen C.P., Juul A. (2022). Dynamic Changes of Reproductive Hormones in Male Minipuberty: Temporal Dissociation of Leydig and Sertoli Cell Activity. J. Clin. Endocrinol. Metab..

[9] O’Shaughnessy P.J., Antignac J.P., Le Bizec B., Morvan M.L., Svechnikov K., Söder O., Savchuk I., Monteiro A., Soffientini U., Johnston Z.C. (2019). Alternative (backdoor) androgen production and masculinization in the human fetus. PLoS Biol..

[10] Dzemaili S., Tiemensma J., Quinton R., Pitteloud N., Morin D., Dwyer A.A. (2017). Beyond hormone replacement: Quality of life in women with congenital hypogonadotropic hypogonadism. Endocr. Connect..

[11] Çiftci N., Akıncı A., Akbulut E., Çamtosun E., Dündar İ., Doğan M., Kayaş L. (2023). Clinical Characteristics and Genetic Analyses of Patients with Idiopathic Hypogonadotropic Hypogonadism. J. Clin. Res. Pediatr. Endocrinol..

[12] Swee D.S., Quinton R. (2019). Congenital Hypogonadotrophic Hypogonadism: Minipuberty and the Case for Neonatal Diagnosis. Front. Endocrinol..

[13] Laitinen E.M., Vaaralahti K., Tommiska J., Eklund E., Tervaniemi M., Valanne L., Raivio T. (2011). Incidence, phenotypic features and molecular genetics of Kallmann syndrome in Finland. Orphanet J. Rare Dis..

[14] Lee H.S., Shim Y.S., Hwang J.S. (2022). Treatment of congenital hypogonadotropic hypogonadism in male patients. Ann. Pediatr. Endocrinol. Metab..

[15] Swee D.S., Quinton R. (2022). Current concepts surrounding neonatal hormone therapy for boys with congenital hypogonadotropic hypogonadism. Expert. Rev. Endocrinol. Metab..

[16] Young J., Xu C., Papadakis G.E., Acierno J.S., Maione L., Hietamäki J., Raivio T., Pitteloud N. (2019). Clinical Management of Congenital Hypogonadotropic Hypogonadism. Endocr. Rev..

[17] Bouvattier C., Maione L., Bouligand J., Dodé C., Guiochon-Mantel A., Young J. (2011). Neonatal gonadotropin therapy in male congenital hypogonadotropic hypogonadism. Nat. Rev. Endocrinol..

[18] Hatipoğlu N., Kurtoğlu S. (2013). Micropenis: Etiology, diagnosis and treatment approaches. J. Clin. Res. Pediatr. Endocrinol..

[19] Rastrelli G., Vignozzi L., Maggi M. (2016). Different Medications for Hypogonadotropic Hypogonadism. Endocr. Dev..

[20] Busch A.S., Ljubicic M.L., Upners E.N., Fischer M.B., Kolby N., Eckert-Lind C., Jespersen K., Andersson A.M., Frederiksen H., Johannsen T.H. (2021). Cohort profile: The COPENHAGEN Minipuberty Study—A longitudinal prospective cohort of healthy full-term infants and their parents. Paediatr. Perinat. Epidemiol..

[21] Lanciotti L., Cofini M., Leonardi A., Penta L., Esposito S. (2018). Up-To-Date Review About Minipuberty and Overview on Hypothalamic-Pituitary-Gonadal Axis Activation in Fetal and Neonatal Life. Front. Endocrinol..

[22] Rastrelli G., Corona G., Mannucci E., Maggi M. (2014). Factors affecting spermatogenesis upon gonadotropin-replacement therapy: A meta-analytic study. Andrology.

[23] Main K.M., Schmidt I.M., Toppari J., Skakkebaek N.E. (2002). Early postnatal treatment of hypogonadotropic hypogonadism with recombinant human FSH and LH. Eur. J. Endocrinol..

[24] Bougnères P., François M., Pantalone L., Rodrigue D., Bouvattier C., Demesteere E., Roger D., Lahlou N. (2008). Effects of an early postnatal treatment of hypogonadotropic hypogonadism with a continuous subcutaneous infusion of recombinant follicle-stimulating hormone and luteinizing hormone. J. Clin. Endocrinol. Metab..

[25] Sarfati J., Bouvattier C., Bry-Gauillard H., Cartes A., Bouligand J., Young J. (2015). Kallmann syndrome with FGFR1 and KAL1 mutations detected during fetal life. Orphanet J. Rare Dis..

[26] Lambert A.S., Bougneres P. (2016). Growth and descent of the testes in infants with hypogonadotropic hypogonadism receiving subcutaneous gonadotropin infusion. Int. J. Pediatr. Endocrinol..

[27] Stoupa A., Samara-Boustani D., Flechtner I., Pinto G., Jourdon I., González-Briceño L., Bidet M., Laborde K., Chevenne D., Millischer A.E. (2017). Efficacy and Safety of Continuous Subcutaneous Infusion of Recombinant Human Gonadotropins for Congenital Micropenis during Early Infancy. Horm. Res. Paediatr..

[28] Papadimitriou D.T., Chrysis D., Nyktari G., Zoupanos G., Liakou E., Papadimitriou A., Mastorakos G. (2019). Replacement of Male Mini-Puberty. J. Endocr. Soc..

[29] Kohva E., Huopio H., Hietamäki J., Hero M., Miettinen P.J., Raivio T. (2019). Treatment of gonadotropin deficiency during the first year of life: Long-term observation and outcome in five boys. Hum. Reprod..

[30] Álvarez Casaño M., López Siguero J.P. (2019). Monitorización del tratamiento del hipogonadismo hipogonadotropo en el lactante [Monitoring of the treatment of hypogonadotropic hypogonadism in the infant]. An. Pediatr..

[31] Avril T., Hennocq Q., Lambert A.S., Leger J., Simon D., Martinerie L., Bouvattier C. (2023). Gonadotropin administration to mimic mini-puberty in hypogonadotropic males: Pump or injections?. Endocr. Connect..

[32] Casarini L., Santi D., Brigante G., Simoni M. (2018). Two Hormones for One Receptor: Evolution, Biochemistry, Actions, and Pathophysiology of LH and hCG. Endocr. Rev..

[33] Liu Z., Mao J., Wu X., Xu H., Wang X., Huang B., Zheng J., Nie M., Zhang H. (2016). Efficacy and Outcome Predictors of Gonadotropin Treatment for Male Congenital Hypogonadotropic Hypogonadism: A Retrospective Study of 223 Patients. Medicine.

[34] Rhys-Evans S., Howard S.R. (2024). Combined gonadotropin therapy to replace mini-puberty in male infants with congenital hypogonadotropic hypogonadism. Ann. N. Y. Acad. Sci..

[35] Stuckey B.G.A., Nolan J.D., Hurley D.M., Martin G.B. (2023). Congenital hypogonadotrophic hypogonadism, induction of minipuberty, and future fertility. Endocrinol. Diabetes Metab. Case. Rep..

